# Australian arm of the International Spinal Cord Injury (Aus-InSCI) community survey: 1. population-based design, methodology and cohort profile

**DOI:** 10.1038/s41393-022-00850-6

**Published:** 2022-09-24

**Authors:** James W. Middleton, Mohit Arora, Annette Kifley, Timothy Geraghty, Samantha J. Borg, Ruth Marshall, Jillian Clark, Andrew Nunn, Anna Ferrante, Christine Fekete, Gerold Stucki, Bamini Gopinath, Ashley Craig, Ian D. Cameron

**Affiliations:** 1 John Walsh Centre for Rehabilitation Research, The Kolling Institute, Northern Sydney Local Health District, St Leonards, NSW Australia; 2 Translational Research Collective, Faculty of Medicine and Health, The University of Sydney, Sydney, NSW Australia; 3 State Spinal Cord Injury Service, Agency for Clinical Innovation, St Leonards, NSW Australia; 4 Spinal Outreach Service, Royal Rehab, Ryde, NSW Australia; 5 Queensland Spinal Cord Injuries Service, Division of Rehabilitation, Princess Alexandra Hospital, Brisbane, QLD Australia; 6 The Hopkins Centre, Griffith University, Brisbane, QLD Australia; 7 South Australian Spinal Cord Injury Service, Central Adelaide Local Health Network, Adelaide, SA Australia; 8 Faculty of Health and Medical Sciences, University of Adelaide, Adelaide, SA Australia; 9 Victorian Spinal Cord Service, Austin Health, Heidelberg, VIC Australia; 10 School of Population Health, Curtin University, Bentley, WA Australia; 11 Swiss Paraplegic Research, Guido A. Zäch Institute, Nottwil, Switzerland; 12 Department of Health Sciences and Medicine, University of Lucerne, Lucerne, Switzerland; 13 Macquarie University Hearing, Macquarie University, Sydney, NSW Australia

**Keywords:** Rehabilitation, Health policy, Quality of life

## Abstract

**Study design:**

Cross-sectional survey.

**Objectives:**

To describe design and methods of Australian arm of International Spinal Cord Injury (Aus-InSCI) community survey, reporting on participation rates, potential non-response bias and cohort characteristics.

**Setting:**

Survey of community-dwelling people with SCI at least 12 months post-injury, recruited between March 2018 and January 2019, from state-wide SCI services, a government insurance agency and not-for-profit consumer organisations across four Australian states.

**Methods:**

The Aus-InSCI survey combined data for people with SCI from nine custodians, using secure data-linkage processes, to create a population-based, anonymised dataset. The Aus-InSCI questionnaire comprised 193 questions. Eligibility, response status and participation rates were calculated. Descriptive statistics depict participant characteristics. Logistic regression models were developed for probability of participation, and inverse probability weights generated to assess potential non-response bias.

**Results:**

1579 adults with SCI were recruited, a cooperation rate of 29.4%. Participants were predominantly male (73%), with 50% married. Mean age was 57 years (range 19–94) and average time post-injury 17 years (range 1–73). Paraplegia (61%) and incomplete lesions (68%) were most common. Males were more likely than females to have traumatic injuries (*p* < 0.0001) and complete lesions (*p* = 0.0002), and younger age-groups were more likely to have traumatic injuries and tetraplegia (*p* < 0.0001). Potential non-response bias evaluated using selected outcomes was found to be negligible in the Aus-InSCI cohort.

**Conclusions:**

The Aus-InSCI survey made efforts to maximise coverage, avoid recruitment bias and address non-response bias. The distributed, linked and coded (re-identifiable at each custodian level) ‘virtual quasi-registry’ data model supports systematic cross-sectional and longitudinal research.

## Introduction

Spinal cord injury (SCI) has far-reaching physical, psychosocial and economic effects not only on the person injured but also people close to them and society more broadly. The International Perspectives on Spinal Cord Injury (IPSCI) report developed by the International Spinal Cord Society (ISCoS) in collaboration with the World Health Organisation (WHO) highlighted a pressing need to improve systematic, routine data collection and increase research on SCI [1]. Understanding the complex interaction between the person with SCI, their environment and participation in the community, as well as the impact of these factors on health and functioning remains a challenge, but important to inform policy and practice changes. Collection of relevant data on functioning, disability and needs of people with SCI across the life span, the ‘lived experience’ of SCI, and the status of SCI-related acute, rehabilitation and community-based systems, provides essential information on what governments, healthcare professionals, rehabilitation centres, community organisations and society in general can do to improve the lives of people with SCI. This may address disparities in their health, functioning, social integration and opportunities.

Large community surveys have previously been undertaken in Canada [2] and Switzerland, the latter as part of ongoing SwiSCI Cohort Study [3–5]. However, until now few studies have comprehensively assessed problems and needs of people with SCI living in the community across different countries [6] and regions, particularly long-term. The International Spinal Cord Injury (InSCI) community survey was instigated in response to IPSCI recommendations as a key first step in gathering internationally comparable data on the ‘lived experience’ of people with SCI and informing development of a new Learning Health System for SCI [7].

There is a lack of large, population-based Australian studies examining the lived experience and most important problems and needs of people with SCI along the continuum of care. This first paper, in a series of three, introduces the methodology of the Australian arm of the InSCI Community survey (known as the Aus-InSCI survey) and characterises the cohort profile. The second paper provides an overview of Aus-InSCI survey results depicting the lived experience, along with learnings and future recommendations. The third paper discusses the drivers of overall quality of life in people with SCI living in Australia.

More specifically, this paper aims to:

1. provide a detailed description of the Aus-InSCI survey design, methods and data linkage processes to obtain a population-based sample
2. report on eligibility, response status and participation (absolute cooperation, contact and response) rates
3. describe sociodemographic and lesion characteristics of the cohort, and evaluate differences in response behaviour (mode and timing), and
4. compare characteristics of participants and non-participants, and evaluate potential non-response bias by developing inverse probability weights accounting for non-response in statistical analyses.

## Methods

The Aus-InSCI survey forms part of a global cross-sectional study to describe the lived experience of people with SCI, within and across countries and corresponding health and social support systems, policies, services, and care. Details of the InSCI survey are described elsewhere [8].

### Study design and participation of data custodians

The Aus-InSCI study combined 11 databases from nine data custodians across four Australian states (New South Wales, Queensland, South Australia and Victoria), creating a representative, population-based, anonymised master database that serves as the sampling frame for individuals with SCI. Data custodians included the specialist SCI clinical services/units in each state, a government insurance agency and three not-for-profit SCI consumer associations. Two other consumer associations were invited but did not participate.

Prior to data collection, the anticipated composition of the target population was considered based on expert opinion and reports from the Australian Spinal Cord Injury Register, a national register of SCI incidences treated in the seven SCI units in Australia [9]. These anticipated characteristics included proportions living in metropolitan and regional/rural settings (70%, 30%); with paraplegia and tetraplegia (50%, 50%); complete and incomplete impairments (40%, 60%); aged <40 years, 40–60 years & >60 years (40%, 30%, 30%), and time post-injury <10 years, 10–20 years, >20 years (33% each).

### Participants

Adults aged 18 years or over, who were residing in the community and at least 12 months post-injury, were able to fill in the questionnaire in English and had either a traumatic injury (e.g., due to motor vehicle crash, fall) or non-traumatic, non-progressive SCI disease or disorder (e.g., from spinal stenosis, infection, vascular accident or primary neurological tumour) were eligible.

Adults with a congenital SCI (such as spina bifida) or neurodegenerative disorders (including multiple sclerosis and amyotrophic lateral sclerosis, or peripheral nerve damage, such as Guillain-Barré Syndrome), those currently receiving acute or subacute care in hospital or unable to complete the survey due to severe cognitive impairments (i.e., severe traumatic brain injury, major mental health condition or dementia) or inability to speak English, were excluded.

### Data linkage and creation of master database

Each data custodian prepared a dataset containing records of all eligible individuals with identifiable information (such as name and date of birth) and sociodemographic and injury-related information. The nine participating custodians prepared a total of 11 datasets, and securely transferred them to a third-party data linkage facility, the Population Health Research Network - Centre for Data Linkage (PHRN-CDL) based at the Curtin University in Western Australia. The PHRN-CDL cleaned, merged and de-duplicated these datasets to create a single master database, which served as the sampling frame for recruitment. The data cleaning phase included the standardisation of data, such as the same codes for gender and the same formats for dates of birth. Missing values or placeholders for missing data were also identified and standardised. The merging and de-duplication of data included a deterministic pass where exact matches were identified. Probabilistic data linkage was then used to determine matches where there were variations in records (e.g., differences due to typographic errors or even changes in addresses). The probabilistic method compares two records and assigns weights based on how closely each field matches. Weights are summed across each field comparison to produce a total weight for the record pair. Only those record pairs with a weight above a certain threshold are accepted as a match. Multiple matching passes ensure that all possible record pairs are assessed. The linkage strategies used in this project were adapted from those used in other multi-jurisdictional data linkage studies, which have been shown to return high-quality linkage results [10, 11]. The master database was then forwarded to the Australian Institute of Health and Welfare (AIHW) in Canberra for linkage with the National Death Index (NDI), identifying individuals who were deceased [12]. AIHW returned the NDI-linked dataset to the PHRN-CDL. A final cleaned and linked master dataset was prepared, assigning a master key identifier with unique national and international IDs and passwords. Eleven re-identifiable datasets containing unique records were then returned to the respective nine data custodians for recruitment. Additionally, a de-identified, population-based master dataset, including basic injury characteristics and National and International IDs and passwords was sent to the national co-ordinating study centre, John Walsh Centre for Rehabilitation Research (JWCRR), Kolling Institute, Sydney.

During the above database handling process, rigorous data management protocols were applied by the PHRN-CDL to protect the privacy and confidentiality of individuals. These include strict data governance procedures covering people, processes and information technology; role separation and restricted data flows to mitigate risks to privacy by limiting access to certain information [13]. The ethically approved record linkage process in Australia was without the specific written consent of each person with SCI and on the basis that this data was believed to be in the public interest and low risk (under Section 95 A of Commonwealth Privacy Act 1988/2014).

### Recruitment and data collection

Eligible individuals were invited to participate by their respective data custodians, with two reminders sent to individuals who had not responded at 3 and 6 months after the initial invitation. At each time point, participants were sent a package, including an invitation or reminder letter, participant information sheet, a blank Aus-InSCI survey (with a unique international ID and a password to access online completion) and a pre-paid self-addressed return envelope. Recruitment was by an opt-out approach. Participants in this study were not under any obligation to complete the questionnaire. Implied consent was used for participants who completed surveys.

The study commenced on 5 March 2018, and recruitment finished on 31 January 2019. Participants could complete the survey as a paper version returned via the pre-paid self-addressed envelope, online by logging into the InSCI website (using their unique Australian ID and password provided to them in the invitation/reminder package) or via telephone interview.

### The Aus-InSCI questionnaire

The InSCI data model, based on the International Classification of Functioning, Disability and Health (ICF) Core Sets for SCI and Rehabilitation, has previously been described [7]. The Aus-InSCI questionnaire is compiled in English, comprising the InSCI module (with 125 questions) and an additional national module, including 68 questions. The InSCI questionnaire includes sociodemographic factors, SCI characteristics, body functions and structures, activities and participation, environmental and personal factors, and health and well-being, and it took between 45–60 min to complete. For more details, see Appendix A of paper 2 of this series [14].

### Statistical analysis

Eligibility, response status and participation rates were described according to the standard definitions of the American Association for Public Opinion Research [15]. Participants’ questionnaire responses were used to describe cohort characteristics. A minimal dataset of core sociodemographic and injury-related information from data custodians on all eligible individuals was used to compare participant and non-participant characteristics. Participation status (participation vs. non-participation) was regressed on a set of sociodemographic and injury characteristics to identify potential predictors for participation using logistic regression analysis, both before and after adjustment for other factors. Odds ratios (OR) and 95% confidence intervals are reported, whereby OR above 1 indicate a higher probability for survey participation and OR below 1 indicate a lower probability of participation.

To correct for potential bias due to unit non-response, logistic regression models for propensity to participate were developed considering age, gender, socioeconomic status, geographical region, recruitment source, injury level and injury duration. Predicted propensities for participation derived from these models were used to generate inverse probability weights, which were then used in subsequent analyses to correct for the potential non-response bias. Reweighted estimates for the percentage of individuals in current paid work and for mean quality of life ratings, modified self-reported Spinal Cord Independence Measure (m-SCIM-SR) total scores [16], and Nottwil Environmental Factor Inventory Short Form (NEFI-S) scores [17] were compared with unweighted estimates, both overall and by gender and lesion level, using survey-weighted generalised linear models. The m-SCIM-SR score used in these analyses involved 12 questions derived from the standard SCIM-SR measure, covering self-care, sphincter management, use of the toilet, and three mobility questions (ability to perform bed-mobility activities unassisted, and degree of independence in transferring from bed to a wheelchair, and in moving moderate distances of 10-100 metres), rescaled to range from 0 (least independent) to 100 (most independent) [16]. The NEFI-S evaluated environmental barriers to participation in society over the past four weeks and was scored between 0 (fewest barriers) and 100 (most barriers) [17]. The self-rated quality of life ratings used were coded from 1 (very poor) to 5 (very good).

Differences in sociodemographic and injury characteristics of participants were examined by recruitment source, including the type of data custodian (consumer organisation, government agency, or SCI unit) and location of data custodian (New South Wales, Queensland, South Australia, or Victoria), and by response characteristics, including speed of response to survey invitations (first three months, next three months, or last four months of data collection period) and response mode (online, telephone, or paper-based).

## Results

**Fig. 1:**
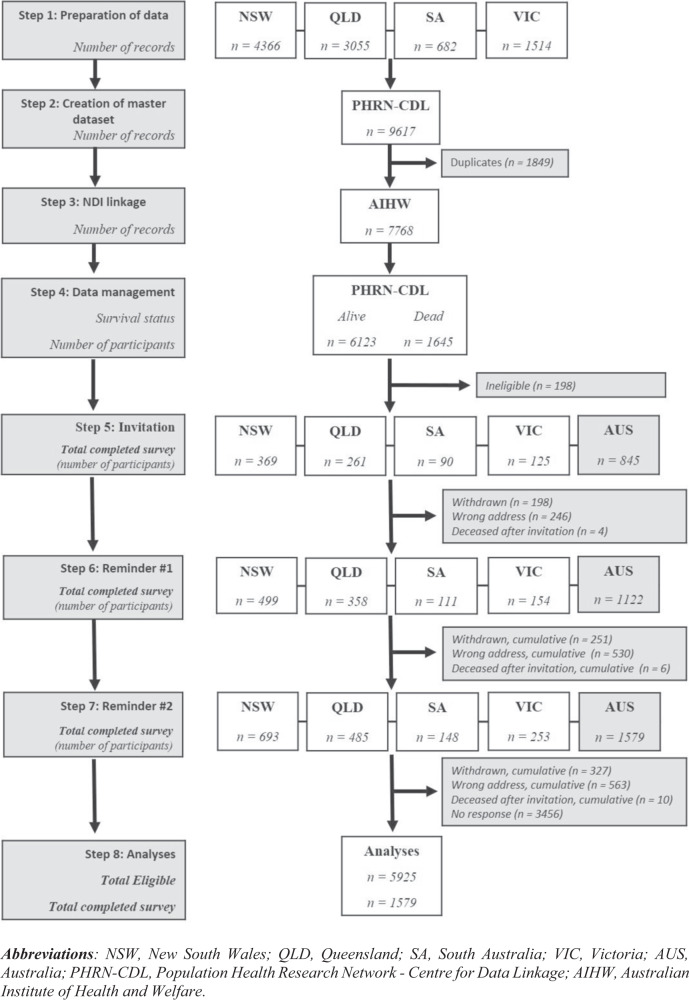
Participant flow diagram. This figure outlines different steps related to data linkage, recruitment process and number of records included at each step.

A total of 9617 records were provided for data linkage, among whom 6123 individuals were alive and sent survey invitations. Of these, 5925 individuals were finally confirmed as eligible, and a study cohort of 1579 participants completed questionnaires (Fig. 1), representing a cooperation rate of 29.4% and a response rate of 26.6% (Table 1). Consumer associations (31.6-33.2%) and the government insurance agency (32%) achieved higher response rates than the state-based specialist SCI services/units (22.4-24.8%).

**Table 1:** Eligibility, response status and participation rates for Aus-InSCI study.

Total invited (*n*)	I + R + NC + NE	6123
Eligible	E = I + R + NC	5925
Not eligible	NE	198
*Reasons for ineligibility:*		
Moved abroad		98
Age below 18 years		12
Medical exclusion criteria		21
Deceased before invitation		66
Duplicate		1
Response status (*n*)		
Participation	I	1579
Non-participation		4346
*Reasons for nonparticipation:*		
Refusal	R	3783
Active refusal		327
Passive refusal		3456
Give up reminding		3446
Deceased after invitation		10
No contact (wrong address)	NC	563
Participation rates (%)		
Cooperation rate	[I/(I + R)]*100	[1579/(1579 + 3783)]*100 = 29.4%
Contact rate	[(I + R)/(I + R + NC)]*100	[(1579 + 3783)/(1579 + 3783 + 563)]*100 = 90.5%
Response rate	[I/(I + R + NC)]*100	[1579/(1579 + 3783 + 563)]*100 = 26.6%

### Comparison of participants and non-participants

Characteristics of all eligible individuals, participants and non-participants, and odds ratios for participation versus non-participation, are presented in Table 2. Participation was more likely among individuals in regional versus metropolitan areas (OR 1.2–1.3), although was less likely for those living in remote versus metropolitan areas (OR 0.7). Participation was more likely for individuals with lengthy (>40 years) versus short (1–5 years) time post-injury (OR 1.5) but was less likely with time post-injury of 11–30 years versus 1–5 years (OR 0.6–0.8). Participation was less likely among individuals with tetraplegia versus paraplegia (OR 0.7). After mutual adjustment between these factors, results were similar, except that the finding of higher participation in regional areas became specific to inner regional versus metropolitan areas, and findings of lower participation for mid-range times post-injury were only significant in the 11–15 year subgroup.

**Table 2:** Characteristics of all eligible individuals with SCI, participants and non-participants, and likelihood for survey participation.

	All eligible individuals (*n* = 5925)	Participants (*n* = 1579)	Non-participants (*n* = 4346)	Unadjusted OR (95% CI) for participation
	*Mean (SD) [Range]*	*Mean (SD) [Range]*	*Mean (SD) [Range]*	*P value*
Age at time of survey (years)	54 (16) [18**–**100^a^]	58 (14) [19–94]	52 (16) [18**-**100^a^]	*p* < 0.0001
SEIFA, IRSAD^b^	5.6 (3.0) [1–10]	5.7 (2.9) [1–10]	5.7 (3.0) [1–10]	*p* = 0.4
Time since injury (years)	17 (13) [1–81]	17 (14) [1–73]	17 (14) [1–81]	*p* = 0.4
	*n (%)*	*n (%)*	*n (%)*	
*Data custodian state*				*p* < *0.0001*
New South Wales	2366 (40)	691 (44)	1675 (39)	1.0 (ref)
Victoria	1079 (18)	251 (16)	828 (19)	0.74 (0.62, 0.87)
Queensland	2000 (34)	481 (31)	1519 (35)	0.77 (0.67, 0.88)
South Australia	471 (8)	147 (9)	324 (7)	1.10 (0.88, 1.37)
*Gender*				*p* = *0.03*
Female	1451 (25)	422 (27)	1029 (24)	1.0 (ref)
Male	4411 (75)	1157 (73)	3254 (76)	0.87 (0.76, 0.99)
*Age group*				*p* < *0.0001*
18–30 years	462 (8)	76 (5)	386 (9)	1.0 (ref)
31–45 years	1411 (24)	246 (16)	1165 (28)	1.07 (0.81, 1.43)
46–60 years	1863 (32)	532 (34)	1331 (31)	2.03 (1.55, 2.65)
61–75 years	1550 (27)	590 (37)	960 (23)	3.12 (2.39, 4.08)
76 years or more	524 (9)	135 (9)	389 (9)	1.76 (1.28, 2.42)
*Injury level*				*p* < *0.0001*
Paraplegia	2728 (54)	936 (60)	1792 (52)	1.0 (ref)
Tetraplegia	2297 (46)	621 (40)	1676 (48)	0.71 (0.62, 0.81)
*Injury duration*				*p* < *0.0001*
1 to 5 years	1103 (20)	353 (22)	750 (20)	1.0 (ref)
6-10 years	1010 (19)	317 (20)	693 (18)	0.97 (0.80, 1.17)
11–15 years	897 (17)	235 (15)	662 (17)	0.75 (0.62, 0.92)
16–20 years	625 (12)	156 (10)	469 (12)	0.71 (0.56, 0.89)
20–25 years	486 (9)	122 (8)	364 (9)	0.71 (0.56, 0.91)
26–30 years	298 (6)	68 (4)	230 (6)	0.63 (0.46, 0.85)
31–35 years	352 (7)	100 (6)	252 (7)	0.84 (0.64, 1.10)
36–40 years	296 (6)	89 (6)	207 (5)	0.91 (0.69, 1.21)
More than 40 years	318 (6)	131 (8)	210 (5)	1.49 (1.15, 1.93)
*Geographical remoteness (ABS)*				*p* < *0.0001*
Major cities	2957 (52)	743 (49)	2214 (53)	1.0 (ref)
Inner regional	1267 (22)	379 (25)	888 (21)	1.27 (1.09, 1.48)
Outer regional	1138 (20)	331 (22)	807 (19)	1.22 (1.04, 1.43)
Remote	172 (3.0)	31 (2.0)	141 (3.4)	0.66 (0.44, 0.98)
Very remote	168 (3.0)	32 (2.1)	136 (3.3)	0.70 (0.47, 1.04)

^a^Age data ranged up to 118 years in eligible non-participants with no obvious cut-off in frequency counts between the plausible and non-plausible values. A threshold of 100 was used as a plausible upper limit, year of birth data implying ages above this threshold was treated as missing data. Sensitivity analysis regarding the choice of 100 as the upper limit of plausible ages for eligible non-participants indicated that ORs strengthen as the cut-off threshold for the oldest group is increased above 100.

^b^SEIFA, IRSAD is the Socio-economic Indexes for Areas, Index of Relative Socio-economic Advantage and Disadvantage.

### Preferred response mode, response times and recruitment sources by participant characteristics

The choice of online response mode was related to younger age, higher socioeconomic status (subjective social position, education and income), metropolitan setting and tetraplegia. Only the youngest age group (18–30 years) preferred the online response mode, while 40–45% of participants with complete tetraplegia or in the highest socioeconomic groups responded online.

Participants with a language other than English spoken at home were more likely than English speakers to respond late in the data collection period. Early responders were more likely to be from older age groups (>60 years) or to have long duration post-injury (>30 years). The latest responders appeared to come from 31–45 years age-group.

Consumer organisations were helpful in picking up participants with very long (≥31 years) time post-injury and complete lesions. Consumer organisations had a higher proportion of participants who were female, receiving day-to-day assistance and of higher educational or socioeconomic status, however, these associations depended on participating state. Government databases containing relatively recent cases of traumatic SCI due to road trauma were helpful in picking up participants from the youngest age group (18–30 years), with short time post-injury (≤5 years and 6–10 years) and with language other than English spoken at home.

Participants recruited by data custodians based in different states displayed different patterns of injury characteristics, socioeconomic status, and proportion from regional versus metropolitan settings.

### Description of cohort

Table 3 displays the sociodemographic and injury characteristics of participants. Participants were predominantly male (73%) with an average age of 57 years (median 59, interquartile range 48–68). Most were living with at least one other adult with or without children (69%), while 23% lived alone. Most lived in metropolitan centres (57%), with 26% in rural centres and 17% in other rural or remote areas. Just over half (55%) had post-secondary education, including 24% with a bachelor or postgraduate degree or equivalent. Nevertheless, 26% were in the lowest category for household income and a further 14% in the next lowest. For self-rated position on the social ladder, 41% were in the lowest four rungs, 27% in the top four rungs, and 32% in the two central rungs.

**Table 3:** Characteristics of the Australian InSCI study population.

	Mean (SD)	[Range]
Age at time of survey (years)	57.5 (14.4)	[19–94]
Age at time of injury (years)	40.5 (17.9)	[1–88]
Time since injury (years)	17.0 (13.7)	[1–72]
Subjective social position^a^	5.0 (2.2)	[1–10]
	*n* (%)	[95% CI]
*Gender*		
Male	1157 (73.3)	[71.0, 75.5]
Female	422 (26.7)	[24.5, 29.0]
*Marital status*		
Single	386 (24.5)	[22.4, 26.8]
Married	791 (50.3)	[47.7, 52.9]
Cohabiting or in a partnership	140 (8.9)	[7.5, 10.5]
Separated or divorced	195 (12.4)	[10.8, 14.2]
Widowed	62 (3.9)	[3.0, 5.1]
*Household composition*		
Living alone	361 (23.0)	[20.9, 25.2]
Living in an institutional setting	55 (3.5)	[2.6, 4.6]
Living with other adults	943 (60.1)	[57.6, 62.6]
Living with kids under 18	72 (4.6)	[3.6, 5.8]
Living with adults and kids under 18	138 (8.8)	[7.4, 10.4]
*Day-to-day assistance*		
Not received	426 (27.2)	[25.0, 29.5]
Received from professionals	325 (20.7)	[18.7, 22.9]
Received from family and/or friends	359 (22.9)	[20.8, 25.1]
Received from family/friends and professionals	457 (29.2)	[26.9, 31.5]
*Highest educational level*		
Primary	61 (3.9)	[3.0, 5.1]
Lower secondary	434 (28.0)	[25.7, 30.4]
Higher secondary	207 (13.4)	[11.7, 15.2]
Post-secondary	292 (18.9)	[16.9, 20.9]
Short tertiary	179 (11.6)	[10.0, 13.3]
Bachelor or equivalent	239 (15.4)	[13.6, 17.4]
Master or equivalent	137 (8.8)	[7.4, 10.4]
*Household income (per annum)*		
Less than $23,660	356 (25.9)	[23.5, 28.3]
$23,712 – $35,672	194 (14.1)	[12.3, 16.1]
$35,724 – $47,268	164 (11.9)	[10.2, 13.8]
$47,320 – $62,556	146 (10.6)	[9.0, 12.4]
$62,608 – $80,496	146 (10.6)	[9.0, 12.4]
$80,548– $100,412	121 (8.8)	[7.3, 10.5]
$100,464 – $123,448	86 (6.3)	[5.0, 7.7]
$123,500– $154,388	51 (3.7)	[2.7, 4.9]
$154,440– $206,908	62 (4.5)	[3.4, 5.8]
$206,960 or more	50 (3.6)	[2.7, 4.8]
*Level/extent of injury*		
Tetraplegia, complete	128 (8.6)	[7.2, 10.2]
Tetraplegia, incomplete	449 (30.3)	[27.9, 32.8]
Paraplegia, complete	362 (24.4)	[22.2, 26.8]
Paraplegia, incomplete	542 (36.6)	[34.1, 39.2]
*Cause of injury*		
*Traumatic*	1306 (83.6)	[81.6, 85.4]
Transport	481 (30.5)	[28.2, 32.8]
Fall more than 1 metre	156 (9.9)	[8.4, 11.5]
Fall less than 1 metre	53 (3.4)	[2.5, 4.4]
Sport	138 (8.7)	[7.4, 10.3]
Leisure	172 (10.9)	[15.0, 18.9]
Violence	17 (1.1)	[0.6, 1.8]
Work-related	116 (7.4)	[6.1, 8.8]
Other traumatic cause	78 (4.9)	[3.9, 6.2]
*Non-traumatic*	257 (16.4)	[14.6, 18.4]
Degeneration	48 (3.0)	[2.2, 4.1]
Benign tumour	34 (2.2)	[1.5, 3.0]
Malignant tumour	10 (0.6)	[0.3, 1.2]
Vascular problem	62 (3.9)	[3.0, 5.1]
Infection	57 (3.6)	[2.7, 4.7]
Other non-traumatic cause	46 (2.9)	[2.1, 3.9]
*Unknown cause*	16 (1.0)	[0.5, 1.7]
*Aboriginal or Torres Strait Islander*		
No	1521 (97.6)	[96.7, 98.4]
Yes	37 (2.4)	[1.6, 3.3]
*Language other than English spoken at home*		
No	1410 (90.0)	[88.3, 91.5]
Yes	157 (10.0)	[8.5, 11.7]
*Living area*		
Australian Capital Territory	16 (1.1)	[0.6, 1.7]
New South Wales	618 (40.6)	[38.0, 43.1]
Northern Territory	6 (0.4)	[0.1, 0.8]
Queensland	480 (31.5)	[29.1, 33.9]
South Australia	141 (9.3)	[7.8, 10.9]
Tasmania	24 (1.6)	[1.0, 2.4]
Victoria	230 (15.1)	[13.3, 17.0]
Western Australia	9 (0.6)	[0.2, 1.2]
*Living place*		
Capital city	517 (34.2)	[31.8, 36.7]
Other metropolitan centre (population > 100,000)	347 (23.0)	[20.8, 25.2]
Large rural centres (population 25,000–99,999)	237 (15.7)	[13.9, 17.7]
Small rural centre (population 10,000–24,999)	153 (10.1)	[8.6, 11.8]
Other rural area (population < 10,000)	126 (8.3)	[7.1, 9.9]
Remote area (population < 5000)	130 (8.6)	[7.2, 10.2]

Missingness ranges between 0% to 6%, except for household income (13%).

^a^Subjective position on social ladder: 1 represents lowest, 10 represents highest.

Paraplegia was more common than tetraplegia, and incomplete lesions more common than complete, with 37% of participants having incomplete paraplegia, 30% incomplete tetraplegia, 24% complete paraplegia and 9% complete tetraplegia. A traumatic cause of injury was listed in 84%, most commonly due to transportation, leisure activities and falls. Traumatic injuries were much more likely to be complete than non-traumatic injuries (37% vs 12%, data not shown). Males were more likely than females to have traumatic injuries (χ^2^ *p* < 0.0001) and complete lesions (χ^2^ *p* = 0.0002, data not shown), and younger age groups were more likely than older groups to have traumatic injuries and tetraplegia (χ^2^ *p* < 0.0001, data not shown).

Mean duration of injury was 17 years (median 13 years, interquartile range 6–25 years). Mean age at time of injury was 40 years (median 38 years, interquartile range 24–55), and was substantially higher on average for participants with non-traumatic injuries (mean 48, median 52, interquartile range 34–64 years) compared with traumatic injuries (mean 38, median 36, interquartile range 23–52 years) (two sample *t*-test *p* < 0.0001). Traumatic injuries due to falls, especially falls from low height, occurred later in life on average than those due to sport and leisure activities, violence and work (mean age at injury for traumatic SCI due to falls from less than 1 metre was 58 years while falls from greater than 1 metre was 47 years, compared to younger ages at injury from sports, leisure, violence or transport between 33-37 years and other work-related causes at 40 years, all *p* < 0.05).

### Correction for unit non-response

Reweighted estimates of mean QoL ratings, m-SCIM-SR scores and NEFI-S scores were similar to estimates that did not adjust for unit non-response, both overall and by lesion level or gender (Table 4). Reweighted estimates of percent in paid work were also similar. One larger change was identified, in the beta coefficient for difference in work participation between men and women, where there was just under 1 SE of change towards less difference after reweighting.

**Table 4:** Correction for unit non-response by inclusion of inverse probability weights in deriving point estimates of selected outcomes.

	Overall % Mean (SE)	Men % Mean (SE)	Women % Mean (SE)	Difference in adjusted model β (SE)	Paraplegia % Mean (SE)	Tetraplegia % Mean (SE)	Difference in adjusted model β (SE)
In paid work							
Unweighted	29.1 (1.2)	31.5 (1.4)	22.6 (2.1)	0.57 (0.15)	28.0 (1.5)	30.9 (1.9)	0.0027 (0.13)
Reweighting 1	32.6 (1.4)	34.7 (1.6)	26.5 (2.6)	0.43 (0.16)	32.1 (1.8)	33.6 (2.1)	−0.095 (0.14)
Reweighting 2	32.8 (1.4)	34.9 (1.6)	26.4 (2.6)	0.42 (0.16)	32.1 (1.8)	33.6 (2.1)	−0.090 (0.14)
Quality of life rating							
Unweighted	3.66 (0.025)	3.66 (0.30)	3.67 (0.05)	0.013 (0.058)	3.67 (0.03)	3.64 (0.04)	−0.027 (0.053)
Reweighting 1	3.67 (0.027)	3.65 (0.032)	3.72 (0.05)	−0.054 (0.059)	3.65 (0.04)	3.69 (0.04)	0.025 (0.056)
Reweighting 2	3.67 (0.027)	3.65 (0.032)	3.72 (0.05)	−0.058 (0.059)	3.65 (0.04)	3.68 (0.04)	0.025 (0.055)
m-SCIM-SR							
Unweighted	61.4 (0.78)	62.3 (0.92)	59.0 (1.47)	3.41 (1.78)	68.1 (0.75)	51.7 (1.47)	−16.3 (1.55)
Reweighting 1	62.6 (0.86)	63.2 (1.02)	60.8 (1.61)	2.46 (1.83)	69.1 (0.78)	53.1 (1.66)	−16.3 (1.77)
Reweighting 2	62.4 (0.87)	63.0 (1.02)	60.6 (1.62)	3.45 (1.78)	69.1 (0.78)	53.0 (1.67)	−16.1 (1.79)
NEFI-S							
Unweighted	33.8 (0.58)	32.8 (0.67)	36.5 (1.11)	−3.24 (1.31)	33.5 (0.74)	34.3 (0.91)	0.25 (1.19)
Reweighting 1	33.7 (0.63)	33.0 (0.74)	35.9 (1.22)	−2.61 (1.38)	33.8 (0.82)	34.1 (1.00)	−0.08 (1.27)
Reweighting 2	33.9 (0.63)	33.2 (0.74)	36.2 (1.23)	−2.82 (1.40)	33.7 (0.82)	34.2 (1.00)	−0.15 (1.27)

Inverse probability weighting for correction of unit non-response.

Reweighting 1: model for the weights used age, gender, SEIFA, remoteness, data custodian. These weights were defined in 5555 (94%) of those eligible and 1516 (96%) of participants.

Reweighting 2: model for the weights used the above variables plus injury level and duration. These weights were defined in 4435 (75%) of those eligible and 1492 (94%) of participants.

Adjusted models are logistic regression models for paid work, normal models for quality of life rating, m-SCIM-SR (modified Spinal Cord Independence Measure for Self Report) and NEFI-S (Nottwil Environmental Factors Inventory Short Form).

## Discussion

In this first paper of the series, we have described the unique design features of the Aus-InSCI survey as the first large survey to examine (and in future follow) the lived experience of people with SCI in Australia. Further, we have reported on participation rates, participant characteristics and differences between respondents and non-respondents. We demonstrate that potential non-response bias is minor in the Aus-InSCI sample, as the inclusion of inverse probability weights did not substantially alter estimates for outcomes of paid work, quality of life, functioning and environmental factors.

The Aus-InSCI design is distinct amongst the twenty-two participating countries [18], as a population-based study, including systematic efforts to maximise coverage, avoid recruitment bias and address non-response bias. The application of the unique data-linkage methodology in this study allows for creation of an anonymized master dataset for use as a sampling frame, combining data from different sources under an ethical framework that preserves privacy. Its design permits removal of people who have died, response tracking and management of reminders. The active role played by SCI consumer associations in the study increased its legitimacy, with higher participation rates when invitations came from consumer associations than SCI services. This may also reflect more recent contact information, a tendency for higher engagement in societal activities and greater openness to contributing to research activities among people who are actively engaged in consumer associations.

The privacy-preserving population-based framework and processes employed ensure that only de-identified data is made available to the researchers and data ownership remains with the separate data custodians. With planning underway to repeat the InSCI survey in 2023, the Aus-InSCI design and methodology has the clear advantage of allowing for (re)identification (by data custodians) and resurveying of previous participants (longitudinal data), as well as initial testing of new samples (creating a new cross-sectional study cohort) in an efficient and systematic way using sequential methods [19]. With the study being repeated over successive timepoints, cohort-sequential, cross-sequential and time-sequential analyses can be applied to obtain longitudinal and contemporary information about the ‘lived experience’ of people with SCI across Australia as a function of age, time post-injury, and cohort. In cross-sectional studies, no differential time (i.e., period) effects can be observed as the data are all collected at one time point. In addition, a cross-sectional method confounds effects of age with cohort differences as the age groups being studied are drawn from different birth cohorts. In contrast, single-cohort longitudinal studies, by definition, cannot discriminate cohort differences, but confound the effects of age changes with changes due to time post-injury. The effect of premature ‘ageing’ on level of function and independence after SCI is recognised to be an increasingly important issue [20], which may be better understood through application of sequential design and analysis methods. In addition, there is the potential for the data-linkage to be extended in future applications to include other sources of secondary information derived from electronic medical record or social services information to answer questions that are in the public interest. For example, the results of Aus-InSCI survey could be linked in future to the Australian Spinal Cord Injury Register [21] for representation, or outcome data collected under the National Disability Insurance Scheme and other schemes for Motor Vehicle and Workers Compensation.

The absolute response rate for current survey was 27%, with a slightly higher cooperation rate (29%) after removal of uncontacted individuals. Response rates for other countries participating in the InSCI survey ranged between 23% and 54% [18]. Comparable response and cooperation rates were seen in countries with similarly performing health systems, such as Germany (32/37%) and the Netherlands (33/34%). The extent to which participants differ from the total population is key to evaluating representativeness of sample, rather than response rate per se [22]. In relation to the Aus-InSCI survey, non-response was related particularly to current age, injury duration, lesion level and rurality, with younger age groups, people with tetraplegia, people between 11–30 years post-injury and those living in remote regions being underrepresented. No difference was seen in socioeconomic disadvantage. Lower participation rates in younger people, as well as those between 11–30 years post-injury, may relate to perceived burden due to survey length, differences in factors such as educational level and work status, which were not available among the non-participants, as well as better perceived health and wellbeing needing less ongoing contact with health services. Reasons for reduced participation rates in social surveys cited by other researchers include poor contact information, concerns about privacy and confidentiality, being over-researched, lack of personal salience, and confusion with telemarketing and other non-scientific campaigns [23, 24].

The final composition of the cohort reasonably reflected a priori expectations based on known sociodemographic and injury characteristics for the prevalent traumatic and non-traumatic SCI population in Australia [9]. The proportion of participants with paraplegia vs tetraplegia, complete vs incomplete impairment, and short versus longer time post-injury were within 10% of a priori expectations, while the proportion of participants in younger age groups were lower than a priori expectations by 19%, and metropolitan vs regional/rural locations by 18%. Differences among these subcategories between participants and non-participants were also generally within 10%, including those based on geographical location, the only exception being a 16% difference in the proportion in younger age groups. Falls from low height were relatively underrepresented as a cause of traumatic SCI, however, when injury mechanism was compared by age grouping, high and low falls were the most common causes in people over the age of 60 years. Similar profiles for injury characteristics of survey participants were reported in Canadian [2] and Swiss [4, 5] SCI studies.

Notably, reweighting for sociodemographic details, remoteness and injury characteristics demonstrated little difference in key outcome variables of modified SCIM score, paid work participation rates, environmental barriers (NEFI score) and ratings of quality of life.

Among countries participating in the InSCI survey, Australia was in the highest quartile of gross domestic product based on purchasing power parity (GDP PPP) [18]. However, the typical gross household incomes of Australian participants with SCI were distinctly lower than typical values for the general Australian population at the time of survey, which were median AUD $1,701 and mean AUD $2,242 per week [25].

Having mixed modes (online, mailed and telephone) for survey completion and sending reminders were important for increasing participation among a diverse population. Surprisingly, most respondents preferred to complete the survey manually rather than electronically, which may reflect demographic and rural disparities in internet use. Use of postal reminders increased response rates considerably. Comparison of early versus late respondents can inform future survey recruitment strategies, with consideration of possible incentives or complimentary approaches to increase participation of underrepresented groups.

This study is not without some limitations, requiring more intensive resources and access to specialised expertise for data-linkage by a respected third-party under strict data governance. It also does not have full national coverage, with Western Australia (11% of population) not participating. Organisational and administrative changes at the SCI Unit in Western Australia (one of the five Australian states with specialised SCI services) during the time when the Aus-InSCI survey was being implemented lead to that state not participating in this first survey, although they intend to participate in the next survey. In addition, our Aus-InSCI sample comprised a majority of traumatic cases, which may reflect the data sources whereby a large number of patients with non-traumatic injuries do not reach specialised SCI care, potentially limiting representativeness and generalisability of results to non-traumatic SCI population. This possible bias can be mitigated in a future survey by linking to data captured in the national rehabilitation medicine integrated outcomes centre.

The possibility of lifespan reduction among some subgroups of people with SCI, such as tetraplegia, could give rise to survivorship bias, which cannot be distinguished using the currently available cross-sectional data. While Aboriginal and Torres Strait Islander background was specifically collected, information about ancestry and cultural background was not otherwise included in favour of questions about other key sociodemographic and socioeconomic characteristics, which are among the most robust determinants of disparities in health outcomes. The systematic collection of core sociodemographic and injury-related variables among eligible non-participants is a clear strength of the study, however, could not extend to collection of in-depth information about living arrangements, partnership status or specific post-SCI issues, such as presence of cognitive impairment or neuropsychological disorders. The self-report nature of information collected in this study could also be prone to reporting bias.

Foremost amongst challenges with data acquisition were differences in the extent and accuracy of data available from diverse health and consumer association sources, with missing or alternative items, different coding with mismatches and problems mapping data between sources, and loss or reduction of information during data harmonisation. Strategies to reduce the unique data disparities between organisations include quality improvement processes, review and standardisation of coding practices, integrating these data into existing operational data flows, building or upgrading information management systems and interoperability between different systems, and staff training. Use of a ‘hierarchy of accuracy’ is helpful when judging data that is conflicting. Oversampling is a strategy that may be used to increase coverage of smaller groups. Finally, for the consumer organisations that did not participate, review of data access processes and protections will help to build trust in data custodians about sharing and releasing data.

## Conclusion

The Aus-InSCI Community Survey represents the largest survey of community-dwelling people with SCI (by a factor of about four, *n* = 1579 versus *n* = 443) ever conducted in Australia. Based on the ICF model, it covers a very broad range of issues and measures related to self-reported health, functioning, social inclusion, economic participation, support needs and quality of life for people with SCI living in Australia; providing a baseline for future comparison within Australia, as well as the opportunity for international benchmarking. This paper provides a potential model for future development of a ‘virtual quasi-data registry’ for research in uncommon conditions, such as SCI.

## Data Availability

De-identified data is available upon request and with permission gained from the Aus-InSCI Community Survey National Scientific Committee.

## References

[1] World Health Organization, International Spinal Cord Society. International perspectives on spinal cord injury. Bickenbach J, Officer A, Shakespeare T, von Groote P, editors. Malta 2013.

[2] Noreau L, Noonan VK, Cobb J, Leblond J, Dumont FS (2014). Spinal cord injury community survey: a national, comprehensive study to portray the lives of canadians with spinal cord injury. Top Spinal Cord Inj Rehabil.

[3] Post MW, Brinkhof MW, von Elm E, Boldt C, Brach M, Fekete C (2011). Design of the Swiss Spinal Cord Injury Cohort Study. Am J Phys Med Rehabil.

[4] Brinkhof MW, Fekete C, Chamberlain JD, Post MW, Gemperli A (2016). Swiss national community survey on functioning after spinal cord injury: Protocol, characteristics of participants and determinants of non-response. J Rehabil Med.

[5] Gross-Hemmi MH, Gemperli A, Fekete C, Brach M, Schwegler U, Stucki G (2021). Methodology and study population of the second Swiss national community survey of functioning after spinal cord injury. Spinal Cord.

[6] Kennedy P, Lude P, Taylor N (2006). Quality of life, social participation, appraisals and coping post spinal cord injury: a review of four community samples. Spinal Cord.

[7] Fekete C, Post MW, Bickenbach J, Middleton J, Prodinger B, Selb M (2017). A Structured Approach to Capture the Lived Experience of Spinal Cord Injury: Data Model and Questionnaire of the International Spinal Cord Injury Community Survey. Am J Phys Med Rehabil.

[8] Gross-Hemmi MH, Post MW, Ehrmann C, Fekete C, Hasnan N, Middleton JW (2017). Study Protocol of the International Spinal Cord Injury (InSCI) Community Survey. Am J Phys Med Rehabil.

[9] AIHW, Tovell A Spinal cord injury, Australia, 2016–17. Injury research and statistics series no. 129. Cat. no. INJCAT 209. Canberra: Australian Institute of Health and Welfare. 2020.

[10] Boyd JH, Randall SM, Ferrante AM, Bauer JK, McInneny K, Brown AP (2015). Accuracy and completeness of patient pathways-the benefits of national data linkage in Australia. BMC Health Serv Res.

[11] Spilsbury K, Rosman D, Alan J, Boyd JH, Ferrante AM, Semmens JB (2015). Cross border hospital use: analysis using data linkage across four Australian states. Med J Aust.

[12] Australian Institute of Health and Welfare. Australian Government. National Death Index. https://www.aihw.gov.au/about-our-data/our-data-collections/national-death-index/about-national-death-index, Accessed on 8-Apr-2022.

[13] Boyd JH, Randall SM, Brown AP, Maller M, Botes D, Gillies M (2020). Population Data Centre Profiles: Centre for Data Linkage. Int J Popul Data Sci.

[14] Middleton JW, Arora M, Kifley A, Clark J, Borg SJ, Tran Y, et al. Australian arm of the International Spinal Cord Injury (Aus-InSCI) Community Survey: 2. Understanding the lived experience in people with spinal cord injury. Spinal Cord. 2022. 10.1038/s41393-022-00817-7. 10.1038/s41393-022-00817-7 PMC9712098 35705701

[15] The American Association for Public Opinion Research. 2016. Standard Definitions: Final Dispositions of Case Codes and Outcome Rates for Surveys. 9th edition. AAPOR. Accessed 24th August 2021.

[16] Fekete C, Eriks-Hoogland I, Baumberger M, Catz A, Itzkovich M, Luthi H (2013). Development and validation of a self-report version of the Spinal Cord Independence Measure (SCIM III). Spinal Cord.

[17] Ballert CS, Post MW, Brinkhof MW, Reinhardt JD (2015). Psychometric properties of the Nottwil Environmental Factors Inventory Short Form. Arch Phys Med Rehabil.

[18] Fekete C, Brach M, Ehrmann C, Post MWM, Stucki G (2020). Cohort Profile of the International Spinal Cord Injury Community Survey Implemented in 22 Countries. Arch Phys Med Rehabil.

[19] Schaie KW (1965). A General model for the study of developmental problems. Psychol Bull.

[20] Hitzig SL, Eng JJ, Miller WC, Sakakibara BM (2011). An evidence-based review of aging of the body systems following spinal cord injury. Spinal Cord.

[21] AIHW: Harrison J, O’Brien D, Pointer S. Spinal cord injury, Australia, 2017–18. Injury research and statistics series no. 136. Cat. no. INJCAT 219. Canberra: AIHW 2021.

[22] Johnson TP, Wislar JS (2012). Response rates and nonresponse errors in surveys. Jama.

[23] Galea S, Tracy M (2007). Participation rates in epidemiologic studies. Ann Epidemiol.

[24] Mitchell S, Ciemnecki A, CyBulski K, Markesich J. Removing barriers to survey participation for persons with disabilities. Rehabilitation Research and Training Center on Disability Demographics and Statistics. Mathematica Policy Research, Inc. Washington, DC: Cornell University; 2006.

[25] Australian Bureau of Statistics, Household Income and Wealth, Australia, 2017-2018. Available at https://www.abs.gov.au/statistics/economy/finance/household-income-and-wealth-australia/latest-release#articles.

